# Trust, affect, and choice in parents’ vaccination decision‐making and health‐care provider selection in Switzerland

**DOI:** 10.1111/1467-9566.13388

**Published:** 2021-11-08

**Authors:** Michael J. Deml, Andrea Buhl, Benedikt M. Huber, Claudine Burton‐Jeangros, Philip E. Tarr

**Affiliations:** ^1^ Institute of Sociological Research Department of Sociology University of Geneva Geneva Switzerland; ^2^ Division of Social and Behavioural Sciences School of Public Health & Family Medicine University of Cape Town Cape Town South Africa; ^3^ Swiss Tropical and Public Health Institute (Swiss TPH) Basel Switzerland; ^4^ University of Basel Basel Switzerland; ^5^ Department of Pediatrics Fribourg Cantonal Hospital Fribourg Switzerland; ^6^ University Department of Medicine Kantonsspital Baselland University of Basel Bruderholz Switzerland

**Keywords:** affect/emotion, choice, complementary and alternative medicine, parenting and health decision‐making, patient–provider interactions, Switzerland, trust, vaccine hesitancy

## Abstract

This article examines the relationships between biomedicine, complementary and alternative medicine (CAM) and parents’ vaccination decision‐making in Switzerland. Our empirical evidence sheds light on an understudied phenomenon—parents switching from one doctor to another provider (often one offering CAM services) around issues that arise during vaccination consultations. This is important to understand since CAM is used by 25%–50% of the Swiss population and is integrated into the Swiss health‐care system when offered by biomedically trained medical doctors with additional CAM training. Qualitative data gathered from in‐depth semi‐structured interviews with parents (*N* = 30) and ethnographic observations of vaccination consultations (*N* = 16 biomedical consultations, *N* = 18 CAM consultations) demonstrate how there was not always a clear‐cut, direct relationship between (non)vaccination and parents’ use of CAM and/or biomedicine. Borrowing from Hirschman (*Exit, voice, and loyalty: Responses to decline in firms, organizations, and states*, Harvard Univ. Press, 1970), we frame our analysis by using the concepts of *exit*, *voice* and *loyalty* to describe parents’ provider selection and vaccination decision‐making process, although only four families in the sample described switching solely because of vaccination‐related issues. Findings add to vaccine decision‐making literature by describing and analysing the underdiscussed provider‐switching phenomenon and by demonstrating the importance of parents’ experiences of trust, affect and choice in vaccination consultations as they pursue the best health outcomes for their children.

**Abbreviations**
CAM: complementary and alternative medicine
FOPH: Federal Office of Public Health
HCPs: health‐care professionals
VH: vaccine hesitancy/vaccine hesitant

## INTRODUCTION

A recurring, yet understudied, topic in *vaccine hesitancy* (VH) literatures involves parents’ transition from seeking health‐care services from biomedical doctors to providers of complementary and alternative medicine (CAM), particularly around the issue of vaccination (Dubé et al., 2016; Navin, 2015; Peretti‐Watel et al., 2019). Peretti‐Watel et al. (2019) describe this selection process with the metaphor of parents looking for ‘a shoe that fits’ (p. 1199). There is a need for further descriptions and detailed analyses of the processes behind this phenomenon, particularly in context‐specific, localized settings.

This article describes and analyses parents’ navigation of vaccination decision‐making in their interactions with, and selection of, biomedical and CAM practitioners in Switzerland. We build upon the growing body of literature on VH and literature describing the important roles played by health‐care professionals (HCPs), particularly doctors, in influencing parents’ vaccination decisions. Our analysis finds support in Hirschman (1970)’s seminal organizational theory text wherein he develops the concepts of *exit*, *voice* and *loyalty*. Our empirical data provide a nuanced picture of parents’ pursuit of ‘the shoe that fits’, by highlighting how they frame vaccination decision‐making and provider selection in terms of trust, which is gained, maintained or lost through issues related to affect and choice.

### Parental vaccine hesitancy and vaccine decision‐making: Trust, affect and choice

Vaccination decision‐making is not a standalone issue to be considered outside of parents’ social contexts (Larson et al., 2014). Ward et al. (2017) identified an assemblage of ‘logics of care’ underpinning these decisions. Such choices are tied into more complex issues related to gendered parenting (particularly mothering), birth experiences, perceptions of parents’ responsibility for children's health and development, and vaccination norms among parents’ various social networks (Poltorak et al., 2005). Other logics may include favouring chemical‐free, ‘natural’, and immunity‐strengthening practices in vaccination decisions (Dubé et al., 2016; Reich, 2016b). Furthermore, researchers have demonstrated how parents’ perspectives on vaccination are not set in stone and that parents may follow various ‘vaccination trajectories’ in their decision‐making process (Wiley et al., 2020).

Common parental concerns about vaccination relate to efficacy, necessity, timeliness, potential side effects and the (un)likelihood of children contracting vaccine‐preventable diseases (Dubé et al., 2013). Such concerns are reflected in the scholarship and research on *vaccine hesitancy* (VH), which MacDonald (2015) has defined as a ‘delay in acceptance or refusal of vaccination despite availability of vaccine services’ (p. 4163). Others have criticized this definition by pointing out that referring to hesitancy as a behaviour (i.e., delay, refusal) is problematic because hesitancy is more of a psychological state than a behaviour (Bedford et al., 2018).

The importance of parents’ trust in institutions and health‐care professionals during their vaccination decision‐making process has been well‐documented. Brownlie and Howson (2005), for example, examine trust as a multifaceted concept which is constructed through practices, different types of knowledge (expert, experiential, etc.), and expressed through ‘leaps of faith’ within complex systems and human anxieties about technologies designed to reduce risk. Qualitative work consistently shows that parents who trust their children's HCPs also trust their vaccination recommendations (Ames et al., 2017; Benin et al., 2006). Brown and Calnan (2012) shed light on the ways the social construct of trust has been evolving through the shifting roles of prescribing professionals (i.e., physicians) as ‘mediators of trust’ since pharmaceutical industries are faceless entities in the eyes of many consumers. Who these mediators of trust are and how trust is constructed are questions that should be further examined in the context of vaccination decisions.

Given the importance of trust in vaccination decision‐making, it is incumbent upon public health authorities and HCPs to understand how trust can concretely be fostered for parents considering their vaccine options. We, here, focus on the emotional side of parents’ health decision‐making for their children, since emotions have been shown to be related to parents’ ‘deliberative’ vaccination choices (Forster et al., 2016, p. 609). We recognize the rich history of the concept *emotion* in the sociological literature, which Bericat (2016) defines as ‘the bodily manifestation of the importance that an event in the natural or social world has for a subject’ (p. 493). We here opt to use the term *affect* by borrowing from Slovic et al. (2005), who define it as ‘the specific quality of goodness or badness (a) experienced as a feeling state (with or without consciousness) and (b) demarcating a positive or negative quality of a stimulus’ (p. S35). Ward (2018), in arguing how trust and emotions are entangled, explains ‘trust is an emotion that is based on an expectation about the future: if you trust a doctor to diagnose an illness or provide childhood vaccinations, you expect that he or she will be able to do this properly’ (p. 719).

Researchers have also pointed to parents’ engagement with the rhetoric of *choice* while they consider vaccinations for their children. These stances emphasize the freedom of autonomous subjects to exercise agency in making personal decisions such as vaccinating one's child, outside the confines of public health or governmental interference (Brownlie & Howson, 2006; Ward et al., 2017). Others have similarly highlighted the relationship between VH and vaccine rejection to Western ideals of *intensive mothering* (Hays, 1996), characterized by investment of upper middle class women's time, energy and resources in the pursuit of the social status of *good mothers* through continuous management and mitigation of risk and health‐promoting activities for children (Lupton, 2011; Reich, 2014; Sobo, 2015). Vigilant parenting practices involve parents viewing themselves as experts about their children and assuming ultimate responsibility, through the choices they make as parents, for their children's wellbeing, independent of doctor recommendations (Attwell et al., 2018; Reich, 2016a).

### Health‐care professionals and parental vaccination decisions: Complementary and alternative medicine

Complementary and alternative medicine's relationship with parental vaccination attitudes and practices has sparked research interest. Browne et al. (2015) have linked negative vaccination attitudes to individuals’ preferences for CAM over conventional medicine, spiritual and intuitive ways of reasoning (as opposed to analytic, cognitive styles), and openness to new experiences. Other researchers have associated parental consultations with CAM practitioners to negative vaccination attitudes and/or lower vaccination uptake than among parents who do not consult CAM providers, however, a systematic review of these studies was unable to establish causal relationships (Wardle et al., 2016). That said, research from a representative sample (*N* = 5200) of Spanish residents shows that although VH was strongly associated with distrust in biomedicine, participants’ trust in CAM was interestingly a weak predictor of VH (Hornsey et al., 2020). Such findings demonstrate how discourses pigeonholing users and providers of CAM as categorically anti‐vaccine are misguided and distract focus from biomedical providers’ responsibilities in earning and maintaining trust.

Social science researchers have described elements of CAM provider encounters with parents that likely appeal to VH parents’ above‐described logics of care. For example, Pedersen (2013) demonstrates how Danish CAM users commonly considered CAM as ‘risk free’ and that it ‘could do no harm’. In a similar study, Pedersen et al. (2016) describe how trust emerges through CAM providers’ active listening, engagement with patients’ bodies and material experiences of the encounter, such as how providers are dressed, the presence of potions, ointments, and decorations, or the use of music during consultations. Dubé et al. (2016)’s research with mothers in Québec, Canada showed how, in pursuit of neutral, balanced information about vaccinations, ‘some vaccine‐hesitant mothers considered that CAM practitioners were more credible than public health authorities because they had “nothing to gain” by dismissing vaccination while governments were perceived as having a hidden agenda’ (p. 416). Others have highlighted the importance CAM users attach to being active participants, exercising ‘do‐it‐yourself’ approaches, and as knowing best how to make judgments about their own health (Attwell et al., 2018).

### Exit, voice and loyalty: Parents as health‐care consumers

We conceptually borrow from Hirschman (1970) to inform our analysis of provider switching behaviours around vaccine decision‐making. In his classic organization theory, political science and economics text *Exit*, *Voice*, *and Loyalty*, Hirschman explains how individuals express (dis)satisfaction with the quality of products or services in free market contexts. He argues that customers have two options for expressing dissatisfaction. The first option is *exit*, whereby customers discontinue purchasing products or withdraw from economic relationships. The second option, *voice*, allows customers to ‘express their dissatisfaction directly to management or to some other authority to which management is subordinate or through general protest addressed to anyone who cares to listen’ (p. 4).

Hirschman argues that both mechanisms are tied to economic and political action, with *exit* anonymously allowing individuals to invoke market forces to prevail without the nuisance of face‐to‐face confrontation. *Voice*, on the other hand, is ‘political action par excellence’ in so far as ‘it implies articulation of one's critical opinions rather than a private, “secret” vote in the anonymity of a supermarket’ (p. 16). *Loyalty* to a product or its provider is a lever which can either mitigate or promote individuals’ propensity to enact *exit* or *voice* options in cases of dissatisfaction. Hirschman contends that when customers are satisfied with service or product quality, *loyalty* retains customers’ business.

Others have expanded upon Hirschman's conceptual framework in analyses of health‐care choices by pointing to issues of patient emotion and satisfaction as determinative factors of patients activating these mechanisms (Bishop et al., 2011; Greener, 2009). Drach‐Zahavy et al. (2017) have focused on how the emotional labour of HCPs largely influences patient satisfaction. That said, Lupton (1997) criticizes economic views of patients as purely rational ‘dispassionate, thinking, calculating’ subjects (p. 374). She convincingly argues in favour of consumeristic understandings incorporating the desires, emotions and needs of patients in patient–doctor relationships. According to Lupton, the emphasis people place upon the affective aspects of health care might lead them to seek treatment from health‐care practitioners outside of biomedicine if their affective needs are not being met. Although Lupton discusses patient decisions for themselves, we here consider parents’ choices for their children.

### The Swiss context

Between 25 and 50% of the Swiss population uses CAM and shows favourable CAM attitudes (Klein et al., 2015; Wolf et al., 2006). Despite the popularity of CAM use and the absence of vaccine mandates in routine settings in Switzerland, childhood vaccination rates remain overall high (i.e., 87%–93% nationally for two doses of mumps, measles and rubella vaccine for 2, 8 and 16 year olds). Depending on the vaccine, these rates have remained relatively stable and steadily increasing over the past 20 years (Swiss Federal Office of Public Health, FOPH (2018a)).

Providers of CAM in Switzerland are often biomedically trained doctors with additional CAM training (Hart, 2017). There is generally equitable access to medical services provided by CAM and biomedical doctors since the Swiss voted in 2009 to integrate CAM into the health‐care system by including reimbursement through basic mandatory health insurance (Saller, 2009). CAM service costs are covered when provided by medical doctors with additional postgraduate training in anthroposophical medicine, Traditional Chinese Medicine (TCM)/acupuncture, homeopathy or phytotherapy (FOPH, 2018b). In Switzerland, there is a higher probability of CAM being used among individuals with chronic illness or self‐reported poor health, women, middle‐aged people and more highly educated individuals (Klein et al., 2015).

### Research questions

Switzerland represents an excellent setting to provide detailed characterizations of the relationships between biomedicine, CAM and parental vaccination decision‐making in a supposed open market of health care. We, therefore, sought to understand, describe and analyse how parents: (1) describe the relationship, if any, between their selection of their children's health‐care providers and their vaccination attitudes and choices, (2) make vaccination and provider‐selection decisions in the context of services being offered by physicians with or without additional CAM training.

## METHODS

This study is embedded within a national research programme on vaccine decision‐making for routine childhood vaccines and the HPV vaccine in Switzerland (Deml et al., 2019a). The original intentions of this qualitative study involved understanding how parents make vaccination decisions and investigating relationships between parents’ use of CAM and vaccine attitudes. However, unexpected results shifted our attention and prompted us to report on an underdiscussed phenomenon—parents switching providers or seeking services from another doctor around the issue of vaccination.

We collected qualitative data in the French‐ and German‐speaking regions of Switzerland between August 2017 and August 2018. We conducted semi‐structured, qualitative interviews with parents and observed vaccination consultations between parents and practitioners of CAM and biomedicine. The parents we observed during consultations were not the parents we interviewed due to practical considerations for ethically and adequately recruiting parents of young children to participate in research at the end of a medical consultation. Quite simply, parents made it clear at the end of vaccination consultation observations that they did not have additional time to answer our research questions during a qualitative interview. We also did not want to insist for follow‐up interviews with observed parents since this likely could have had an impact on the doctor–parent relationship.

We recruited by sending study materials to potential participants via participating CAM and biomedical providers and through snowball and convenience sampling with interviewed parents. Given the gendered nature of parental health decision‐making, the majority of our sample was composed of mothers. We purposively sampled more parents reporting CAM use than those only consulting with biomedical practitioners and more VH parents (i.e., those expressing concerns about vaccine efficacy, necessity and/or safety) than vaccine‐accepting parents. We observed vaccination consultations with providers we had previously interviewed (Deml et al., 2019b, 2020), during consultations where vaccinations were likely to be discussed for the first time.

Interviews with parents were audio‐recorded and transcribed verbatim. The interview questions related to family backgrounds, parents’ roles in health choices, children's health, parents’ use of CAM and biomedicine, and parents’ vaccination decision‐making process. We asked parents for copies of their children's vaccination certificates and discussed vaccination status during interviews. We piloted interview guides and revisited them iteratively for clarity. During observations, we documented notes in field journals, which we then wrote into narrative accounts.

Since several authors with different disciplinary backgrounds coded the collected data in German and French, we opted to use the Framework Method to guide our analyses of interview transcripts and observations (Gale et al., 2013). Initial thematic analysis brought us to here specifically focus on parents’ selection of practitioners of CAM and/or biomedicine and influences on parents’ vaccination perspectives (Braun & Clarke, 2006). We used MAXQDA software to code segments of text from the transcripts and narrative accounts of observations.

The local ethics committee approved the study. We obtained informed consent from all participants. We translated quotes from interviews and observations into English. Doctors mentioned specifically with a pseudonym are study participants. We use pseudonyms for all participants. The data that support the findings of this study are available on request from the corresponding author. The data are not publicly available due to privacy or ethical restrictions.

## RESULTS

We interviewed 30 parents (French‐speaking region: *N* = 13, German‐speaking region: *N* = 17) from 26 families. Eight parents were interviewed as couples. We interviewed more mothers (*N* = 24) than fathers (*N* = 6). Parents’ ages ranged from 26 to 55 years (average ~37 years). The number of children per family ranged from one to five (average ~2 children). Most parents had attained education at a bachelor's degree level or higher. We observed a total of 34 vaccination consultations (*N* = 18 CAM consultations with five providers, *N* = 16 biomedical consultations with six providers).

In seven families, at least one child had received none of the recommended vaccines.

In 11 families, children had been partially vaccinated or in an individualized/delayed fashion. In eight families, the children had been vaccinated according to official recommendations. Parents reported using CAM in 21 families. Four families described switching from biomedical doctors to CAM doctors due solely to vaccination‐related issues, several others discussed switching doctors for other reasons, and we observed several parents discussing doctor switching during consultations.

Parents’ vaccination and doctor‐selection decisions are further detailed in the following sections. We first discuss parents’ vaccination provider‐selection choices *vis*‐*à*‐*vis* their relationships with CAM and biomedicine. Second, we show how trust in providers, which was negotiated through parents’ experiences of affect and choice, played determinative roles in parents’ vaccination and provider‐selection decisions.

### Relationships between CAM, biomedicine and (non)vaccination

Participants did not always discuss a direct, linear relationship between consultations with CAM or biomedical providers and their vaccination decisions. Several parents reported choosing doctors based upon recommendations from social networks and positive or negative communicative experiences with HCPs. Others chose doctors based purely on geographical proximity and convenience. We, here, discuss some of the patterns involving parents’ choices of CAM or biomedical practitioners in relationship to vaccination decisions.

During interviews, parents described preconceived notions about biomedicine's relationship with favourable vaccine attitudes and CAM’s relationship with critical vaccine attitudes. Mrs. Galland explained why she did not consult with CAM providers, ‘In general, I think they're all against vaccination. So, I don't really need to see them to know their opinion’. Others described biomedical doctors’ roles in vaccinating with public health goals in mind. Mrs. Diesbach explained how she thought biomedical doctors wanted to ‘absolutely vaccinate’ because ‘doctors’ roles include eradicating disease’.

For vaccine‐accepting parents, the norm was to consult with biomedical paediatricians for routine health check‐ups. These parents described the vaccination decision as a normal step in their children's health care. Mrs. Piccard explained why she had had her son vaccinated, ‘My brothers and I were all vaccinated, and everything was fine. There wasn't really a particular reason that pushed us to vaccinate’.

Some parents made a conscious choice to consult with CAM providers because of their own VH or intentions to not follow vaccination recommendations. One explanation involved parents’ wishes to avoid potential confrontations with, and disapproval from, biomedical HCPs. Mrs. Humbert described having chosen a homeopathic paediatrician who was open to non‐vaccination, explaining that her choice was, ‘especially about that’. Mrs. Heer discussed a similar thought process, ‘I definitely like it when someone has a bit of an alternative background. (…) I simply knew that I had to have a paediatrician who was a bit critical about vaccinations or could accept non‐vaccination’.

Some parents did not link their choice of CAM paediatricians to their vaccination attitudes. Rather, they sought care from CAM providers because parents associated them with ‘natural, chemical‐free’ approaches. Interestingly, we witnessed several parents who were not familiar with the CAM practices or services of the providers they were seeking. Such cases involved parents looking for options outside of traditional, biomedical approaches, without specifically knowing what they were searching for in terms of available CAM services and approaches. For example, Mrs. Sandoz explained, ‘I was looking for a paediatrician who also offered alternative medicine because I’m not so in favour of medication. I prefer both my own and my child's immunity to develop on their own before taking medicine or ingesting chemicals. (…) So, I looked around. I didn't know much about anthroposophy. I read a little bit about it, and it suited me’.

#### Public health framings versus desires for individualization

Parents were aware of vaccination being promoted by public health authorities as a normative medical practice. For example, Mrs. Piccard, who vaccinated according to official recommendations and expressed no VH, noted how her paediatrician had presented vaccinations as a standard protocol, ‘You can sense that it's organized (…) and that it's planned out, even for parents who have not yet accepted vaccination’. Mrs. Chappuis criticized public health authorities’ presentation of vaccination, linking her scepticism and desire for balanced presentation of ‘the facts’ to her higher education, “The Swiss Federal Office of Public Health is pro‐vaccine. They say that you need to vaccinate. For me, I don't know if it's because I pursued higher education, but I like being able to weigh the pros and cons. I like when all sides are presented to me. I don't like just receiving one side of the story’.

Vaccine‐hesitant parents expressed a desire for individualized approaches to vaccination, such as considering each vaccine individually for their children. These parents often wanted more specific information about vaccine‐preventable diseases and the ‘true risks’ of vaccinating or not in Switzerland. Others questioned HCPs’ uncritical vaccination convictions. Mrs. Zurbrügg, a paediatric nurse and mother to five partially vaccinated children, mentioned how her children's first paediatrician tried to ‘sell’ vaccination as a universal approach: ‘[He] talked very disrespectfully about parents who did things differently, like not vaccinating. That always bothered me because nothing is 100% certain’. This doctor's critical attitudes towards parents not following vaccination recommendations were among several reasons why Mrs. Zurbrügg's family switched from this paediatrician to one with CAM training.

### Gaining, maintaining and losing trust through affect and choice

Bolstering previous research, participants’ trust in their HCPs was clearly a major determinative factor in participants’ decision‐making processes (Benin et al., 2006). Many parents cited trust in providers as being one of the most essential aspects for them to meaningfully engage with health information during medical consultations. During interviews and observations, trust, which was gained, maintained and lost around issues of affect and choice, was evidently important for parents both in terms of their vaccination attitudes and the selection of their children's HCPs.

Parents’ expectations for trusting relationships with their children's doctors were not uniform. Some parents trusted doctors enough to delegate the vaccination decision to them or to follow doctors’ suggestions to vaccinate according to official recommendations. Others viewed doctors as health consultants with whom they could discuss their vaccination options, with the parents making the final choice. Some parents had already decided how they wanted to vaccinate before consulting and hoped doctors would respect their choices. Several VH parents described having established lasting relationships with their children's biomedical doctors. In these cases, paediatricians, without insisting too heavily on the issue, had either convinced parents to vaccinate more than they originally intended or were willing to accept parents’ decisions to delay or not vaccinate.

#### Affect: Emotions and social proximity

Parents commonly described experiences with HCPs in emotional terms. They cited affect, or their general sense of comfort or discomfort, as important elements of clinical encounters. Mrs. Piccard described her trusting relationship with her son's paediatrician, simply stating that she had a ‘good feeling’ about him. Conversely, some parents discussed negative emotions and affect experienced in interactions with their children's doctors. Several parents expressed a desire for closer social proximity with their children's providers as a means of improving their experience of affect in clinical encounters.

Mrs. Kugler described how she had switched from seeing a biomedical doctor to consulting with Dr. Heffelfinger (anthroposophic medicine) following a disagreement about the timing of her daughter's vaccination. She explained how the first doctor pushed for Algifor^®^ (ibuprofen) whenever her 2.5‐year‐old daughter had a fever. During a check‐up with this doctor, her daughter was feverish and recovering from an infection. Mrs. Kugler explained losing trust when the doctor insisted on vaccination, ‘[She] was very sick again with an extremely high temperature. Again, the remedy was Algifor. The doctor added, “We should start vaccinating soon. (…) It's a classic fever. We can easily vaccinate. It's not too bad at this age.” (…) I felt we were no longer in good hands and switched to Dr. Heffelfinger’.

Ms. Besse decided to consult with a homeopathic paediatrician because her son's first biomedical paediatrician had referred to her as an ‘unfit mother’ and mentioned her son dying as a possible consequence of non‐vaccination. At first, the original paediatrician was accepting of Ms. Besse's wishes to not vaccinate. However, the doctor changed her mind after talking to the son's father. Ms. Besse explained,

“I switched paediatricians recently (…). [The first one] had been very open to my choice to not vaccinate, but then, the father talked to her about it again. At our last check‐up, she said to me, ‘But you don’t realize, he could die!’ That really upset me because, while I accept that a paediatrician can disagree with me, she shouldn’t make me feel guilty. It’s not the role of a doctor. I need someone with whom I am comfortable.”

She elaborated on her decision to change doctors, ‘I don't want to have my stomach in knots every time I see her because I have certain ideals!’ She considered sending the paediatrician a letter to explain her departure because, during their tense exchange, Ms. Besse was shocked, emotional, and ‘did not have the guts’ to say something. At the time of the interview, she had not sent the letter. She later found a new homeopathic paediatrician after perusing a Facebook group moderated by vaccine‐sceptical mothers in Switzerland. She explained how group members circulated lists of doctors, commonly CAM providers, who were open to non‐vaccination.

During a vaccination consultation with Dr. Buchman (TCM/acupuncture), we observed a mother discuss difficulties finding an HCP with whom she could satisfactorily discuss vaccination. After initially vaccinating her son as recommended, she described how he cried more often. With each subsequent vaccination, he had additional symptoms, such as vomiting, fever and sometimes being unresponsive. She explained to Dr. Buchman how the first biomedical paediatrician had not been supportive when she mentioned these symptoms, which made her to feel ‘left alone’, which led her to find a second paediatrician. Her son's non‐vaccination became a point of tension with the second paediatrician, who told the mother that not vaccinating was ‘irresponsible and harmful’, which both disappointed her and made her lose trust. She explained to Dr. Buchman how she had considered going to a shared paediatric CAM practice of ‘well‐known non‐vaccinators’. However, since she was considering some vaccinations, she needed a provider who could advise her.

This mother's recollection of being called an ‘irresponsible mother’ echoes what other parents described feeling when other people evaluated their vaccine‐related choices. Several participants described how being criticized for their children's health‐care choices was, by extension, a criticism of themselves as parents. Others were comfortable facing such critiques. Mrs. Schmied, mother to an unvaccinated 6‐month‐old daughter, recounted how, although most people aware of her decision were neutral, she heard criticism from one colleague, ‘Someone from work said, “No, I find that irresponsible.” (…) I was not really bothered [by the criticism] because we really thought this through and stand behind our decision’.

Several parents desired being considered on the same social level as doctors, explaining how the social distance in biomedical provider offices could be off‐putting and distract from the parent–provider relationship. Mrs. Chappuis explained her preference for her children's homeopathic paediatrician after having made the transition from a biomedical doctor, ‘There's something so human about him. (…) It's kind of a silly detail, but he doesn't wear the white doctor coat. It's like you're talking to a peer, like someone who is on your level. (…) It's like we're having a coffee together. There's no judgement. It's really pleasant’.

Similar to other research findings (Hertig et al., 2014), many parents wished to interact with their practitioners not only as practitioners but as fellow parents. They described wanting to know how their children's HCPs vaccinated their own children. Mrs. Lopez, for example, had asked her CAM practitioner about his own parenting choices, ‘The doctor who practices alternative medicine told me that it was the parents’ choice. Afterwards, I asked him if he vaccinated his daughters. He told me that he did’. Some parents sought vaccination advice from their midwives who had children in instances when their child's doctor was not a parent. Mrs. Godet, for example, was interested in what her midwife thought because she had the ‘motherly element’ that her paediatrician did not.

#### Choice

Discussions about parental decisions were often framed against debates about vaccine mandate laws in Switzerland's neighbouring countries Italy, France and Germany. During interviews with parents, and in observations of medical consultations, choice was discussed in relationship to the Swiss context, where vaccination is voluntary. Many parents viewed the ability to choose vaccinations independently from medical and governmental recommendations positively.

Parents commonly discussed doctors’ respect of their choices as an important factor for establishing trusting relationships. Mrs. Martin, a mother of three unvaccinated children, described how her biomedical paediatrician had earned her trust:

“[The paediatrician] is a good listener. She respects my choices. That’s not always the case with doctors. For that, I thank her. We continue going to see her because we’re happy. When I told her that we weren’t going to vaccinate, she made her arguments. She made sure that I had understood the consequences of my decision.”

Mrs. Goff, a mother of two partially vaccinated children, described her relationship with her children's biomedical paediatrician, ‘It's nice that there wasn't any pressure. She didn't push me to do anything. I know that she is in favour of vaccination, but she doesn't force. She lets parents have a certain amount of autonomy. We are free in our decision‐making’.

From observation notes, we describe below how two undecided parents explained to Dr. Welty (anthroposophic paediatrician), why they chose him for their 4‐week‐old baby. At this point, they were finalizing a 15‐min discussion about vaccination:

“Dr. Welty said he had not solved their problem. The mother said he did not have to. The doctor told her he could not make the decision for them. She replied by explaining how they had chosen to see him specifically because he did not pressure parents in either direction (observation notes).”

Despite vaccination being voluntary, some parents perceived doctors’ abilities to force patients to vaccinate. Mrs. Chappuis, for example, explained how she felt forced by her daughter's paediatrician:

“She forced me to get vaccinated against the flu and whooping cough, but I’m absolutely against the flu vaccine. I had my little 3‐week‐old baby in my arms, and the doctor said, ‘You know, if she gets the flu, she can die. If she gets whooping cough, she can die, too. You’re not vaccinated against these diseases.’ (…) She said to me, ‘If you don’t do it, your child is going to die.’ What’s a mother supposed to do? You don’t want your child to die.”

This issue culminated in Mrs. Chappuis's choice to leave her daughter's paediatrician and seek cares from a homeopathic paediatrician. While the use of coercion in the above‐mentioned case is debatable, it was in hindsight perceived as such by Mrs. Chappuis due to the doctor's evocation of her daughter's potential death. Mrs. Chappuis described how her children's homeopathic paediatrician afterwards earned her trust by not taking her ‘for an idiot’ and ‘taking the time to have a discussion’. She explained how the paediatrician engaged in the vaccination consultation without judgment, ‘He told us right off, “I happily vaccinate. I am willing to not vaccinate if you don't want to. There is no judgement. You decide.” (…) We really made an informed choice. It was a real choice. It wasn't imposed upon us’.

## DISCUSSION

This article has described and analysed parents’ selection of their children's health‐care providers and vaccination decision‐making at the intersections of CAM and biomedicine in Switzerland. Many VH and vaccine‐rejecting parents demonstrated ‘logics of care’ (Ward et al., 2017) which were enmeshed with parents’ vaccination and provider‐selection decisions. These logics included neoliberal, intensive parenting practices of those pursuing the social status of *good parents* (Deml et al., 2020; Hays, 1996; Reich, 2014). Other logics denoted the importance of ‘natural’, ‘chemical‐free’ regimens involving minimal Western medicine. Similar to the findings of Attwell et al. (2018), parents did not always describe a linear relationship between their use of CAM or biomedicine and (non)vaccination. Similarly, parents in this study did not describe a clear‐cut causal relationship between the choice of their children's doctors and their vaccination attitudes and choices. These discussions were rather enmeshed with parents’ depictions of biomedicine and public health authorities as being influenced by the pharmaceutical industry and profit seeking. Some also associated public health authorities and pharmaceutical companies with biased information in favour of vaccination and as promoting *one*‐*size*‐*fits*‐*all* vaccination approaches. VH parents perceived CAM providers, on the other hand, as offering a space where parents could seek out ‘neutral’ information sources, would not be unduly pressured in vaccination decisions and would be accompanied in their children's health care, regardless of vaccination choices.

Hirschman (1970)’s concepts *exit*, *voice* and *loyalty* are useful for understanding the phenomenon involving parents’ transition from seeking health‐care services from biomedical practitioners to those offered by CAM providers. This conceptual framework could also serve as a useful heuristic for HCPs to better engage with VH parents so as to allow patients space to *voice* their concerns, retain *loyalty* through trusting relationships and avoid patients *exiting* pre‐established relationships with providers who may serve as long‐term trustworthy sources of vaccination information.

Our findings have shown that the enactment of *exit* and *voice* options generally revolved around issues related to trust, choice and affect vis‐à‐vis CAM and biomedicine in clinical encounters. Such results provide qualitative evidence supporting Hornsey et al. (2020)’s assertion that ‘rather than being “pulled” toward vaccine hesitancy because of trust in CAM, people seem to be “pushed” into vaccine hesitancy via *mis*trust of conventional medicine’ (p. 5, emphasis in original).

As a means of expressing disagreement or dissatisfaction, some parents *exited* their relationship with their children's biomedical providers. Parents who chose to *exit* relationships with biomedical doctors did not necessarily do so as a result of overt epistemological differences between themselves and their children's biomedical providers. Several vaccine‐sceptical parents had even maintained relationships with their children's biomedical paediatricians, despite parents’ non‐adherence to official vaccination schedules. It should be noted, however, that as part of the provider selection process, VH parents in search of the ‘truth’ about vaccination were sometimes sceptical about embracing CAM as a valid option due to preconceived notions about CAM providers being categorically anti‐vaccine.

Parents’ ability to enact the *voice* option to express dissatisfaction was rarely evoked in parents’ discussions of clinical encounters or during consultation observations. This might be due, in part, to patients’ fear of being labelled as ‘difficult’ when expressing disagreement in physician–provider interactions which have been characterized as being composed of differential power balances between patients and doctors (Dubé et al., 2016).

Research does show, however, that anti‐vaccine and vaccine safety advocacy groups provide platforms for parents to utilize *voice* options for expressing dissatisfaction with vaccination consultations or vaccinations themselves (Blume, 2006; Navin, 2015; Sobo et al., 2016). Several parents explained how they had discussed negative experiences in clinical encounters around the issue of vaccination, or had heard similar testimonies, with those in their social networks.

*Loyalty* between parents and their doctors was primarily contingent upon the existence of a trusting relationship. As shown with the example of Mrs. Martin's relationship with her unvaccinated children's biomedical paediatrician, parents’ loyalty to children's doctors did not necessarily depend on sharing similar epistemologies about medicine and vaccination, nor on agreement about a correct course of action for children's health care. Rather, it depended more on trust, which was experienced in terms of affect and choice.

Vaccine‐accepting, fully vaccinating parents expressed trust, and by extension, loyalty to their paediatricians. This is not surprising since there were likely few opportunities for *epistemic friction* (Navin, 2015), or potential for conflict or tension about what counts as legitimate evidence upon which decisions can be made, during their vaccination consultations. VH parents likewise expressed trust towards their CAM providers. This is not surprising either, as previous qualitative research has shown that CAM provider approaches in Switzerland likely appeal to the above‐described logics of care of VH parents by framing vaccination choices at individual and family levels and taking time to understand parents’ wishes, involving them in vaccination decisions, and taking their concerns seriously (Deml et al., 2019b).

Although parents’ enactment of *exit*, *voice* and *loyalty* options points to patterns of parents’ expression of (dis)satisfaction with vaccination consultation services, the evidence we present here cannot attest to the overall prevalence of such behaviours. Of the 26 families interviewed in our non‐representative sample, four recounted having actively made the biomedical‐to‐CAM switch solely due to vaccination‐related issues. Others made the switch due to issues related to trust, affect and choice. Our sampling strategy also might have biased our focus on parents’ positive encounters with CAM providers because parents who previously had negative interactions with certain HCPs might have been keen to share these experiences and have their stories heard. Future quantitative work could examine these patterns on a larger scale and more systematically examine these issues in relation to socio‐demographic variables, as our sample was primarily composed of middle and upper middle class mothers.

The concepts *exit*, *voice*, and *loyalty* are perhaps overly simplistic for health‐care decision‐making. Although some parents explained how they had *exited* from their relationship with biomedical providers, it would be a stretch to say that they *exited* biomedicine completely. In other words, despite having exited specific relationships with biomedical providers, parents reported maintaining consultations with biomedical practitioners with no CAM training and/or use of non‐CAM medical products and therapies. Parents who used CAM therapies or services after exiting relationships with certain biomedical providers described how they nonetheless retained a relationship with biomedicine for other health‐related issues, such as for consultations with biomedical providers or consumption of biomedical products. As previously mentioned, parents’ use of *voice* did not always take place during clinical encounters. If researchers wish to pursue parental voice around vaccination (dis)satisfaction, future attention will benefit from focus on parents’ social networks, parental use of the Internet, particularly forums where one can create and upload content, and focus on health social movements. Finally, the concept of *loyalty*, and the idea of *being loyal* to one's HCP, might be too strongly connoted if we wish to use it as a proxy for trusting relationships.

## CONCLUSIONS

Our findings provide several practical implications for HCPs who regularly counsel VH parents. Previous research has shown how such clinical encounters can elicit dilemmas, challenges and dissatisfaction for providers of biomedicine (Deml et al., 2020; Kempe et al., 2015; Philpott et al., 2017). From a public health perspective, public health authorities have recognized that HCPs ‘remain the most trusted advisor and influencer of vaccination decisions, and they must be supported to provide trusted, credible information on vaccines’ (WHO, 2019). Some of this support will come from further understandings of VH and vaccine‐rejecting parents’ rationales and parents’ potential to enact *exit*, *voice* and *loyalty* options. Despite these commonly described phenomena in other research, it is difficult with this study sample and data analysis to establish a ‘universal algorithm’ (Larson et al., 2014, p. 2155) regarding relationships between use of CAM/biomedicine, provider selection and vaccination decisions.

First, HCPs could benefit from understanding that parents who express concerns about vaccination are not categorically anti‐vaccine, do not simply lack information and that their questioning is in search of information in the best interest of their children. Most parents who did not vaccinate according to the official schedule reported not being opposed to the idea of vaccinating their children and, similar to Reich (2018)’s findings, prided themselves in their questioning of recommendations as part of good parenting practices.

Second, HCPs who are dismissive or who do not engage with parents’ VH miss an opportunity for conducive dialogue that acknowledges parents’ commitment to their children's health. Most parents reported seeking out the ‘truth’ about vaccinations, the diseases they protect against and the ‘real risks’ they incur by choosing to vaccinate or not. Similarly, HCPs who counsel CAM‐oriented parents do not necessarily need to align themselves with parents’ inclination towards natural, chemical‐free and anti‐Western medication stances. As we have documented here, several parents reported not being fully committed to CAM as their only health‐care option. Some parents even noted that they did not fully understand certain CAM approaches but appreciated having options for what they perceived as natural, alternative and complementary approaches to biomedicine. Similar to Attwell et al. (2018)’s assertion, we suggest that HCPs could, in encounters with VH patients interested in CAM, ‘adopt a tone of curiosity and partnership, seeking to better understand the core concerns with vaccination and seeking whether there is any room for change in position or compromise’ (p. 113).

Third, biomedical HCPs might underestimate their symbolic roles in representing biomedicine and their influence on parents’ trust in biomedicine and, by extension, vaccination. Such a claim is supported by Hornsey et al. (2020)’s findings that people's distrust of biomedicine is likely a larger obstacle to vaccine acceptance than their trust in CAM. Parents’ framings of their interactions with their children's HCPs demonstrated the importance of ‘good feelings’ with providers for the establishment of trusting and long‐lasting relationships. For many parents, their children's paediatricians are among the first professionals with whom they engage as they begin making health‐care decisions for someone other than themselves—their children.

It is important for researchers and clinicians to understand what parents perceive of as the quality of the services that biomedicine and CAM can offer and what brings health‐care consumers to enact mechanisms of *exit*, *voice* and *loyalty* around the issue of vaccination. As we have shown, the quality of vaccine consultation services for parents was generally experienced through affect and perceptions of choice, which built trust with providers who recognize that parents are seeking the best health outcomes for their children.

## AUTHOR CONTRIBUTIONS

**Michael J. Deml:** Conceptualization (lead); Data curation (lead); Formal analysis (lead); Investigation (lead); Methodology (lead); Project administration (lead); Writing‐original draft (lead); Writing‐review & editing (lead). **Andrea Buhl:** Conceptualization (supporting); Data curation (equal); Formal analysis (supporting); Methodology (supporting); Validation (supporting); Writing‐original draft (supporting); Writing‐review & editing (supporting). **Benedikt Huber:** Conceptualization (supporting); Formal analysis (supporting); Investigation (supporting); Methodology (supporting); Project administration (supporting); Resources (supporting); Writing‐review & editing (supporting). **Claudine Burton‐Jeangros:** Conceptualization (supporting); Formal analysis (supporting); Funding acquisition (supporting); Investigation (supporting); Methodology (supporting); Supervision (lead); Writing‐review & editing (supporting). **Philip E. Tarr:** Conceptualization (lead); Data curation (supporting); Formal analysis (supporting); Funding acquisition (lead); Investigation (supporting); Methodology (supporting); Project administration (lead); Resources (lead); Supervision (lead); Writing‐original draft (supporting); Writing‐review & editing (supporting).

## Data Availability

The data that support the findings of this study are available on request from the corresponding author. The data are not publicly available due to privacy or ethical restrictions.
